# PPARγ Agonistic Activity of Mimulone and Diplacone Encapsulated in Liposomes and Cyclodextrin Complexes

**DOI:** 10.1002/open.202500209

**Published:** 2025-07-31

**Authors:** Daniela Nykodýmová, Lenka Molčanová, Jan Kotouček, Josef Mašek, Jakub Treml

**Affiliations:** ^1^ Department of Molecular Pharmacy Faculty of Pharmacy Masaryk University Palackého tř. 1946/1 612 00 Brno Czech Republic; ^2^ Department of Natural Drugs Faculty of Pharmacy Masaryk University Palackého tř. 1946/1 612 00 Brno Czech Republic; ^3^ Department of Pharmacology and Toxicology Veterinary Research Institute Hudcova 296/70 621 00 Brno Czech Republic

**Keywords:** cyclodextrins, geranylated flavanones, liposomes, nanoparticles, peroxisome proliferator‐activated receptor gamma

## Abstract

The therapeutic application of flavonoids is limited by their low solubility, bioavailability, and metabolic stability. This study evaluates the peroxisome proliferator‐activated receptor gamma (PPARγ) agonistic activity of two geranylated flavonoids from *Paulownia tomentosa*, mimulone and diplacone, and compares the efficacy of different nanoparticle delivery systems, including liposomes and cyclodextrins, in preserving their biological activity. Using the PPARγ CALUX reporter gene assay, it is shown that mimulone dissolved in DMSO and incubated with cell culture activates the PPARγ pathway, resulting in 2.97‐fold and 3.9‐fold increases in luciferase activity at concentrations of 5 and 2.5 μM, respectively. Diplacone, however, shows significant cytotoxicity, with an average cell viability of about 10% at 10 μM. Encapsulation in anionic, cationic, and neutral liposomes results in a significant reduction of biological activity of both flavonoids, with the best formulation (anionic liposomes) preserving only 54% of mimulone's activity. In contrast, hydroxypropyl‐β‐cyclodextrins (HP‐β‐CDs) retain up to 91.5% of mimulone's biological activity and significantly improve the viability profile of diplacone, maintaining cell viability at ≈100%. The performance of the HP‐β‐CDs can be attributed to their ability to form stable inclusion complexes with hydrophobic molecules. These results suggest that cyclodextrin‐based delivery systems might effectively address solubility and stability challenges associated with flavonoid therapy.

## Graphical abstract

Encapsulation of the geranylated flavanones mimulone and diplacone in hydroxypropyl‐β‐cyclodextrins (HP‐β‐CDs) and liposomes is evaluated for their peroxisome proliferator‐activated receptor gamma agonistic activity. While liposomes reduce efficacy, HP‐β‐CDs preserve up to 91.5% of mimulone's activity and mitigate diplacone's cytotoxicity, highlighting cyclodextrins as promising flavonoid carriers.

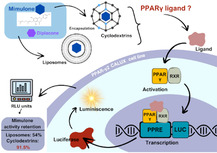

## 1 Introduction

Flavonoids are polyphenolic natural compounds with diverse biological activities and their therapeutic potential is well‐documented, including evidence of benefits in metabolic and inflammatory disorders. However, wide clinical application has been limited by some of their properties, such as low solubility, bioavailability, and metabolic stability. The low water solubility and rapid metabolic elimination of flavonoids necessitate the development of novel delivery systems to improve their therapeutic efficacy.^[^ ^1^ ^]^ Many flavonoids have shown a potential to influence glucose metabolism by modulating the peroxisome proliferator‐activated receptor gamma (PPARγ).^[^ ^2^ ^]^ Previous studies have demonstrated that geranylated flavonoids from *Paulownia tomentosa* Steud. (Paulowniaceae), such as mimulone and diplacone, exhibit potent biological activities, including the inhibition of protein tyrosine phosphatase 1B and α‐glucosidase.^[^ ^3^ ^]^ Based on these results, we investigated whether these compounds can also modulate glucose metabolism via activation of the PPARγ pathway.

PPARs are ligand‐inducible transcription factors that play crucial roles in glucose and lipid metabolism, adipogenesis, and anti‐inflammatory responses.^[^ ^4^ ^]^ There are three isoforms of PPARs: PPARα, PPARβ/δ, and PPARγ.^[^ ^5^ ^,^ ^6^ ^]^ As nonsteroidal nuclear receptors, PPARs are located predominantly in the nucleus, where they form heterodimers with the retinoid X receptor (RXR).^[^ ^7^ ^]^ In their ligand‐free state, PPAR‐RXR heterodimers are bound to corepressor proteins and remain inactive. Upon binding a ligand, a conformational change releases the corepressors, switching the complex to its active state, where it binds to specific DNA sequences called peroxisome proliferator response elements and regulates gene transcription.^[^ ^6^,^8^ ^]^ Activation of PPARγ isoform by specific ligands leads to changes in gene expression that improve insulin sensitivity, fatty acid storage, and glucose homeostasis, making it an important therapeutic target for type 2 diabetes and related metabolic syndromes. While synthetic PPARγ agonists, such as rosiglitazone (a member of the thiazolidinedione (TZD) class), are effective in enhancing insulin sensitivity, TZDs are associated with notable side effects, including cardiovascular risks and weight gain, which have limited their use in long‐term therapy.^[^ ^2^ ^,^ ^9^ ^,^ ^10^ ^]^ The need for safer alternatives has increased interest in identifying natural product agonists and partial agonists that may offer similar benefits with reduced adverse effects. Some flavonoids have shown potential as selective PPARγ modulators (SPPARMs), which may activate PPARγ pathways with partial agonist activity, thereby reducing unwanted side effects seen with full agonists like rosiglitazone.^[^ ^2^ ^]^

Liposomes are highly effective drug delivery systems for flavonoids, capable of improving their solubility, stability, and bioavailability. Recent research has made significant progress in this field, providing innovative solutions for efficiently delivering these bioactive compounds in various applications.^[^ ^11^, ^12^ ^–^ ^13^ ^]^ The interaction and encapsulation of the flavonoids and liposomal particles involve hydrophobic interactions within the lipid bilayer and hydrophilic interactions through hydrogen bonding with the polar head groups of the lipids at the membrane interface.^[^ ^14^ ^]^ These interactions significantly influence the encapsulation efficiency (EE) and release rates of flavonoids from the liposomes. However, overly strong interactions between flavonoids and the liposomal structure can present a limitation, potentially reducing therapeutic efficacy. To address this limitation, we explored cyclodextrins as an alternative drug delivery system. In addition to liposomes, cyclodextrins have emerged as another promising platform for encapsulating flavonoids to address their inherent challenges of poor solubility, stability, and bioavailability. Recent studies have focused on the interactions between flavonoids and cyclodextrins, leveraging the unique molecular structure of cyclodextrins to form inclusion complexes with these bioactive compounds.^[^ ^15^, ^16^, ^17^, ^18^ ^–^ ^19^ ^]^

The aim of this study was to evaluate the PPARγ‐agonistic activity of mimulone and diplacone in vitro and to compare the efficacy of different nanoparticle delivery systems in maintaining their biological activity. In particular, the main objective was to identify the most effective nanoparticle system—among anionic, cationic, and neutral liposomes and cyclodextrins—for encapsulating these geranylated flavonoids and enhancing their bioavailability and stability.

## 2 Results and Discussion

### 2.1 Nanoparticle Analysis

The characterization of the nanoparticles, as determined by dynamic light scattering (DLS) and ultraviolet‐visible (UV–VIS) spectroscopy, is summarized in **Table** **1**. UV–VIS spectroscopy further confirmed the encapsulation of the active compound in the nanoparticles, with the corresponding absorbance peaks used to calculate the EE values. The methodology took advantage of the poor water solubility of the active compound, as mimulone does not exhibit UV–VIS absorbance when unsolubilized. This inherent property ensured that only the solubilized fraction of the compound contributed to the UV–VIS measurements, enabling accurate determination of EE. Furthermore, any unsolubilized aggregates were removed by filtration to eliminate potential interference. The DLS characterization of the liposomes revealed that the average hydrodynamic radius for all liposomal samples, except for the blank neutral liposomes, was ≈154 nm. The blank neutral liposomes exhibited a relatively higher hydrodynamic radius of 194 ± 2 nm. All liposomal samples demonstrated low polydispersity index (PDI) values below 0.15, indicating a narrow size distribution. The distribution analysis, intensity, and number exhibit the narrow profiles of the particle size distribution (the data are available in Appendix A, Figure S1–S5, Supporting Information). The detailed distribution data show that the liposomal samples maintained a consistent and uniform size range, further confirming the reliability and stability of the formulations under the test conditions. The *ζ*‐potential for neutral liposomes remained consistent regardless of the presence or absence of mimulone. In contrast, anionic liposomes (AL) with high mimulone concentration showed a slight increase in *ζ*‐potential from −26.1 ± 2.0 mV for empty liposomes to −23.3 ± 1.8 mV for high mimulone concentrations. For cationic liposomes, the *ζ*‐potential values were 18.0 ± 1.2 mV at low mimulone concentrations and 14.9 ± 1.3 mV at high mimulone concentration, indicating a decrease in the surface charge compared to the empty liposomes, where the *ζ*‐potential value was 18.5 ± 0.9 mV. This suggests that a portion of the active compound could be bound to the surface of the particles.

**Table 1:** Characterization of nanoparticles by DLS including hydrodynamic radius (Z‐average), PDI, concentration (particles mL^−1^), and ζ (zeta potential) measured in mV. The concentration refers to the final concentration of mimulone, with 51 and 204 μg mL^−1^ representing low and high concentrations, respectively, in the case of liposomes. In the case of cyclodextrins (HP‐β‐CDs), the low concentration represents 51 μg mL^−1^ and high (*) 510 μg mL^−1^ of mimulone and diplacone, respectively. The encapsulation efficiency (EE%) was determined from the UV–VIS data.

Sample	Concentration	Z‐Average [d nm]	Polydispersity index (PI)	Concentration [particles mL^−1^]	*ζ* [mV]	EE [%]
Neutral liposomes	Low	156± 3	0.085 ± 0.029	9.12 ± 1.48·10^10^	2.1 ± 0.2	27
	High	168 ± 1	0.079 ± 0.019	3.01 ± 0.06·10^11^	1.4 ± 0.8	77
	Blank	194 ±2	0.104 ± 0.018	3.64 ± 0.18·10^10^	1.3 ± 0.3	–
Anionic liposomes	Low	153 ± 1	0.127 ± 0.003	2.89 ± 0.39·10^11^	−26.6 ± 0.6	42
	High	145 ± 1	0.138 ± 0.013	4.06 ± 0.48·10^11^	−23.3 ± 1.8	60
	Blank	152 ±1	0.123 ± 0.006	3.33 ± 0.08·10^11^	−26.1 ± 2.0	–
Cationic liposomes	Low	166 ± 1	0.105 ± 0.013	2.44 ± 0.05·10^11^	18.0 ± 1.2	82
	High	161 ± 1	0.112 ± 0.007	3.55 ± 0.13·10^11^	14.9 ± 1.3	51
	Blank	167 ± 1	0.098 ± 0.016	2.32 ± 0.17·10^11^	18.5 ± 0.9	–
HP‐β‐CD–mimulone	Low	3 ± 0	0.242 ± 0.005	5.71 ± 3.17·10^17^	–	101
	High*	3 ± 0	0.237 ± 0.003	4.98 ± 1.35·10^17^	–	52
	Blank	2 ± 0	0.166 ± 0.015	1.40 ± 0.31·10^18^	–	–
HP‐β‐CD–diplacone	Low	2 ± 0	0.203 ± 0.003	2.05 ± 2.20·10^18^	–	91
	High*	12 ± 8	0.323 ± 0.026	1.07 ± 0.89·10^18^	–	33

In the case of hydroxypropyl‐β‐cyclodextrins (HP‐β‐CDs), the particle size was consistently uniform at ≈3 nm in diameter across all measured samples. However, the PDI was relatively higher, around 0.2, indicating the presence of some larger entities. The higher PDI in the samples containing mimulone or diplacone indicates that the presence of an active compound contributes to the formation of these aggregates. The blank samples had a lower PDI of 0.166 ± 0.015. The distribution analysis (the data are available in Appendix A, Figure S4–S5, Supporting Information) shows that while larger particles were present, the majority of the particles remained uniformly small. This suggests that HP‐β‐CD forms primarily small particles with a minor population of larger aggregates.

**Figure 1:**
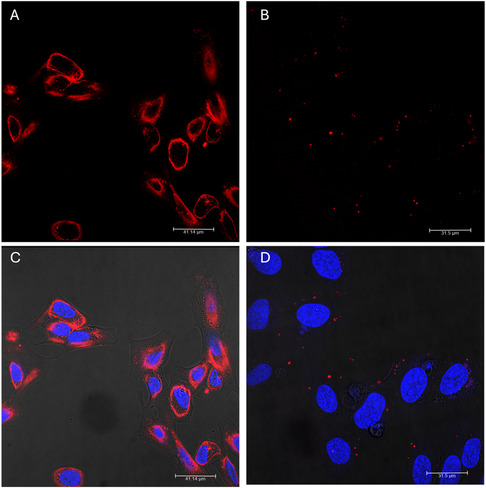
Confocal fluorescence microscopy images of PPAR‐γ2 CALUX cells after 8 h of incubation with neutral and cationic liposomes containing 0.1 mol% of the fluorescent probe 18:1 Liss Rhod PE. A,C) Neutral liposomes consisted of EPC/Chol./DC‐Chol./DSPE‐PEG‐2000 (55/40/25/5); B,D) cationic liposomes were composed of EPC/DC‐Chol (80/20) : (A,B) Liposomes within the cells, showing the distribution and localization of the fluorescently labeled liposomes; (C,D) merged images combining liposome (red) and nuclei (blue) fluorescence signals.

To visualize the uptake of neutral and cationic liposomes, the fluorescent probe Liss Rhodamine was incorporated into the membranes of empty liposomes (without mimulone). From images A and C in **Figure** **1**, it is evident that after 8 h of incubation, a strong fluorescent signal was observed for liposomes with a zeta potential close to 0 mV. This indicates significant cellular uptake for these neutral liposomes. In contrast, for the test cationic liposomes shown in Figure 1B,D, with a zeta potential of ≈18 mV, the uptake by cells was noticeably lower compared to the PEGylated liposomes. Despite the positive surface charge, which generally promotes interaction with negatively charged cell membranes, the cationic liposomes did not exhibit the same level of cellular uptake as the PEGylated counterparts. Although AL were not visualized in this study, the primary purpose of the visualization tests was to confirm that the reduced biological activity of mimulone was not due to a lack of liposome entry into the cells. The results demonstrated that, even with cationic and PEGylated liposomes, which showed low biological activity for mimulone, there was significant particle uptake by the cells following incubation. This indicates that the liposomes were indeed internalized, suggesting that the reduced biological activity of mimulone is not due to insufficient cellular uptake.

### 2.2 Cytotoxicity Assay

**Figure 2:**
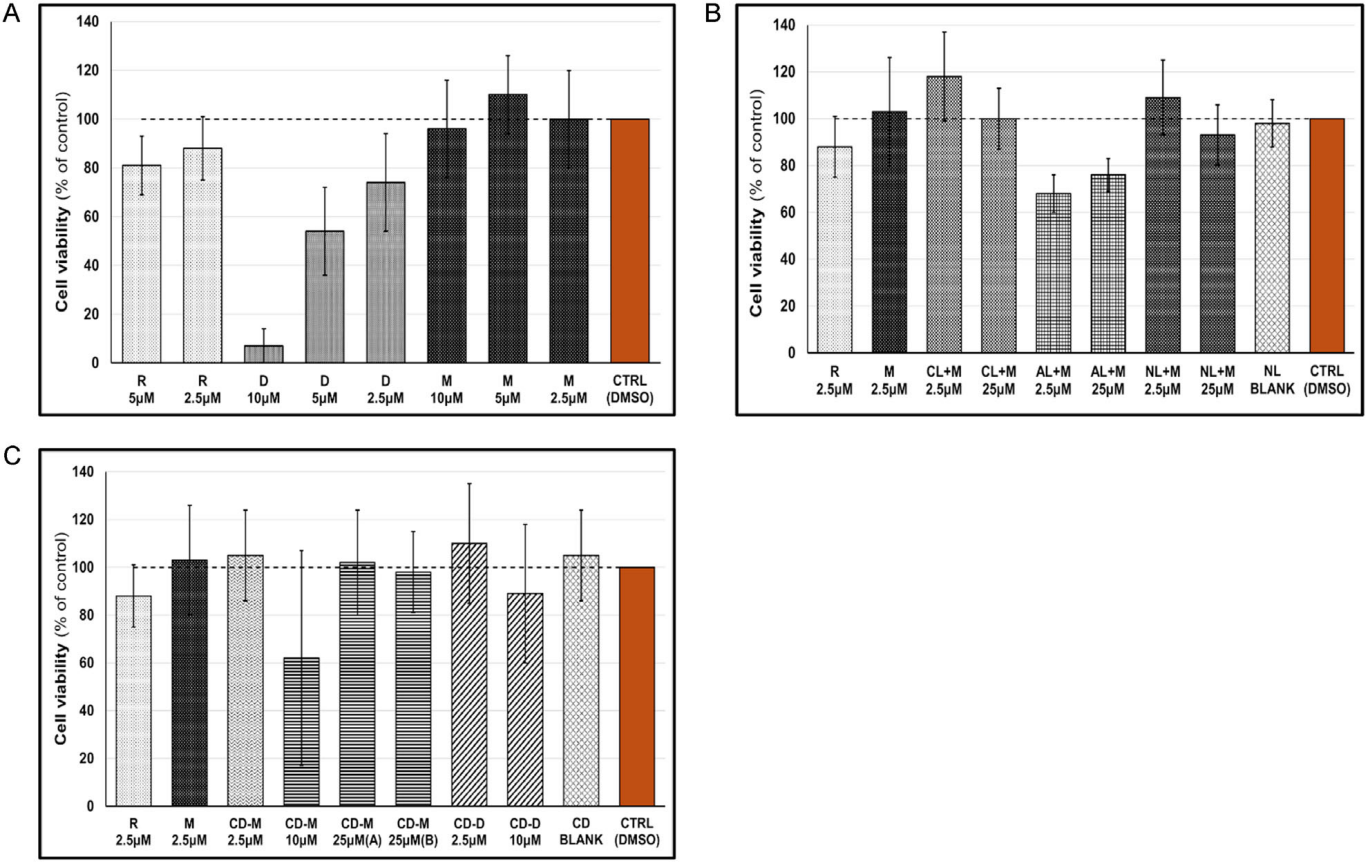
The effect of the TC on the viability of PPAR‐γ2 CALUX cells. The cell viability was measured using the WST method and assessed for free compounds and nanoparticle combinations. A) TC dissolved in DMSO. B) TC encapsulated into liposomes. C) TC encapsulated into HP‐β‐cyclodextrins. R = rosiglitazone; M = mimulone; D = diplacone. Liposomes: cationic (CL), anionic (AL), and neutral (NL); CD refers to HP‐β‐cyclodextrins. The letters (M, D) indicate specific combinations with the test compound; the values are the exact concentrations of the test compound used. Two CD‐M 25 μM combinations differ in CD composition: 10 mg mL^−1^ (sample A) and 100 mg mL^−1^ (sample B). The results are expressed as the mean ± SEM (*n* = 3), measured at least in tetraplicate and compared to the negative control group (DMSO; set as 100%).

The ability of the test compounds (TC) to affect cell viability (cytotoxic effect) was assessed using the water‐soluble tetrazolium salt (WST‐1) method, and the results were compared between the compounds alone and the compounds incorporated into nanoparticles. The aim was to determine whether the encapsulation process of the TC could influence (or potentially reduce) their cytotoxic effect. The WST‐1 assay results on PPAR‐γ2 CALUX cells indicate significant differences in cell viability between the TC and their encapsulated forms. As shown in **Figure** **2**A, mimulone dissolved in DMSO maintained cell viability at ≈100% across all concentrations tested, similar to the control, and indicating no significant cytotoxic effects. In contrast, diplacone exhibited greater cytotoxicity, with an average cell viability of about 10% at 10 μM. Rosiglitazone also demonstrated a decrease in cell viability but still preserved its high biological activity as a full PPARγ agonist in subsequent experiments. Figure 2B shows that AL caused the greatest reduction in cell viability among all of the liposome formulations tested. Nevertheless, the biological effect of encapsulated mimulone on activation of the PPARγ pathway was the highest with AL. In Figure 2C, all cyclodextrin formulations, including their combinations with the TC, maintained cell viability around 100%. This improvement in viability profile was observed for all concentrations of diplacone when it was analyzed as cyclodextrin‐encapsulated diplacone versus pure diplacone. The cyclodextrin‐encapsulated mimulone combination (CD‐M, 10 μM) was an exception, showing a lower average viability and a higher standard deviation compared to other combinations, with no clear explanation for this variability. These observations emphasize the varying impacts of nanoparticle systems on cell viability and their potential to influence the biological activity of the TC.

### 2.3 PPARγ Agonistic Activity of the TC (without Encapsulation)

**Figure 3:**
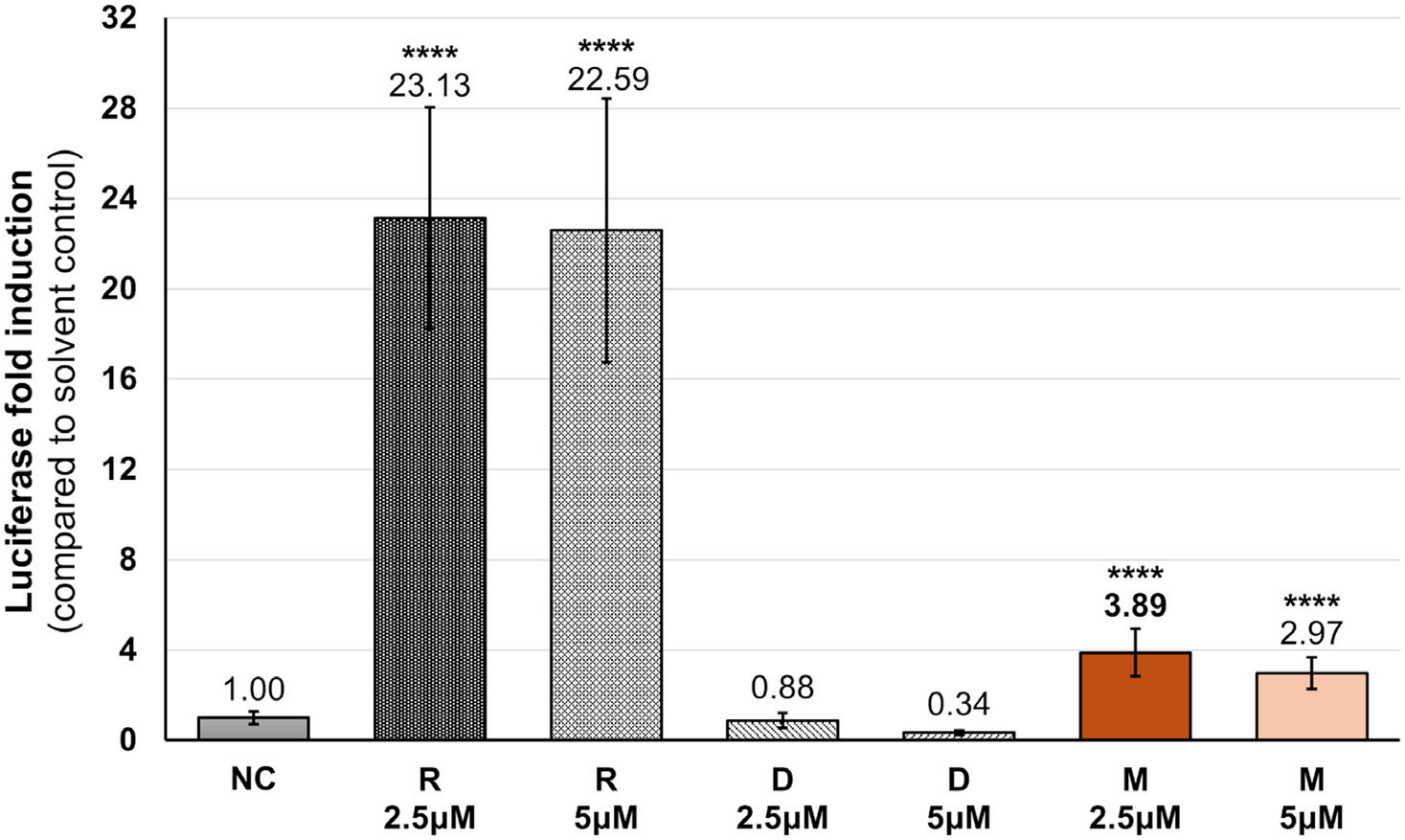
Effect of mimulone (M) and diplacone (D) on activation of the PPARγ pathway. The luciferase activity was measured by the CALUX reporter gene assay in the PPARγ2 CALUX cell line and expressed as fold induction compared to the negative control (i.e., the solvent). Rosiglitazone (R) was used as the standard, DMSO served as the negative control (NC). Values refer to the exact compound concentrations used. The results are expressed as the mean ± SEM (*n* = 3), measured in tetraplicate. Statistical analysis is based on the Kruskal Wallis test followed by the Bonferroni correction for multiple tests. Asterisks indicate a significant difference from the solvent control: *p < 0.05; **p < 0.01; ***p < 0.001; and ****p < 0.0001.

First, we evaluated the ability of the compounds to activate the PPARγ pathway independently at two concentrations: 2.5 and 5 μM. Rosiglitazone was used as a positive control at the same concentrations as the TC. The results of these experiments are summarized in **Figure** **3**, with the luciferase activity expressed as a fold change relative to the negative control (DMSO).

Our findings indicate that mimulone exhibited PPARγ agonistic activity at both concentrations: 5 μM (2.97‐fold) and 2.5 μM (3.9‐fold), both with statistical significance (*p* ≤ 0.0001). Given that the maximum response compared to rosiglitazone at a concentration of 2.5 μM was only 16.8%, we suspect that mimulone acts as a partial agonist in the observed cellular pathway or that activation occurs through other ligand‐independent mechanisms. In contrast, diplacone showed no statistically significant effect at either concentration.

**Figure 4:**
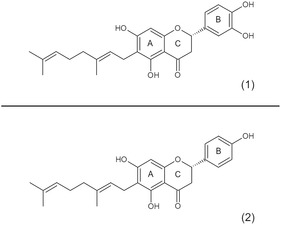
Structures of the TC 1) diplacone and 2) mimulone. The letters A, B, and C denote aromatic and heterocyclic rings according to the conventional nomenclature used for flavonoid scaffolds.

The chemical structure of mimulone, in particular the presence of a hydroxyl group at the C‐4 position on the B‐ring, likely accounts for its observed biological activity (**Figure** **4**). This feature distinguishes mimulone from diplacone, which has two hydroxyl groups at the ring B. Liang et al. emphasized the importance of hydroxyl groups at positions A‐5, A‐7, and B‐4 for PPARγ activation. This matches the mimulone substitution pattern, and they also suggested that an additional hydroxyl group at B‐3 could reduce PPARγ activation. This is consistent with our results and the inactivity observed for quercetin and diplacone, both of which contain a B‐3 hydroxyl group.^[^ ^20^ ^]^ In contrast, another study reported that myricetin, which has additional hydroxyl groups at B‐3 and B‐5, is a more effective PPARγ ligand than kaempferol, which has only a B‐4 hydroxyl group.^[^ ^21^ ^]^ That prenyl group at C‐6 may also have an effect on the mimulone activity is supported by a study of sanggenon‐type flavanones that found the presence of a prenyl group at C‐6 is crucial for PPARγ agonistic effects.^[^ ^22^ ^]^

The structure of mimulone resembles other prenylated flavanones, such as bavachinin, which also showed substantial PPARγ agonistic activity.^[^ ^23^ ^]^ This study aligned with previous findings in that it confirmed that the prenyl group position C‐6 of the A‐ring is crucial for PPARγ agonistic activity. But it also indicated that a methoxyl group at C‐7 increases PPARγ agonist activity, whereas hydroxyl groups, such as these in mimulone, reduce it. However, our results do not support this finding. Mimulone achieved a 3.9‐fold activation at a concentration of 2.5 μM (equivalent to 16.8% of the maximal response of 2.5 μM rosiglitazone), compared to bavachinin's 13.12‐fold activation at 25 μM (19.1% of the maximal response of 5 μM rosiglitazone), whereas the concentration of the test compound was five times as higher as that of the positive control.

There is evidence that PPARγ can be activated through both ligand‐dependent and independent mechanisms. The compounds that stimulate PPARγ in the desired manner, that is, less effect than synthetic agonists and greater effect than weak agonists, are referred to as partial agonists.^[^ ^24^ ^]^ Moreover, structural differences among PPARγ modulators significantly impact their agonistic behavior. Fisetin, for example, acts as a full agonist probably because of its specific structural features.^[^ ^25^ ^]^ Fang et al. demonstrated that kaempferol and quercetin act as partial PPARγ agonists, with maximal responses of less than 45% and 20%, respectively, compared to rosiglitazone.^[^ ^26^ ^]^ This aligns with our findings, where the maximal response of mimulone is less than that of rosiglitazone, suggesting a similar partial agonistic mechanism. Similarly, it has been reported that flavonoids extracted from *Melissa officinalis* and *Matricaria chamomilla/Matricaria recutita* flowers behave as partial agonists for PPARγ by activating it with a half‐maximal effective concentration (EC_50_) of 86 mg mL^−1^ and had 26% of maximal potency as compared to rosiglitazone.^[^ ^24^ ^,^ ^27^ ^,^ ^28^ ^]^ On the contrary, the competitive binding assays conducted by Beekmann et al. showed that quercetin and kaempferol, despite increasing PPARγ‐mediated gene expression, are not direct agonists of the receptor.^[^ ^29^ ^]^ Their action likely involves receptor activation by endogenous agonists and an increase in PPARγ receptor levels. Additionally, the activation mechanisms of hesperidin and morin provide parallels to mimulone's activity. Hesperidin involves both PPARγ‐dependent and independent mechanisms to exert its effects,^[^ ^30^ ^]^ while morin binds to PPARγ and acts as a moderate ligand.^[^ ^31^ ^]^ These findings suggest that the partial agonistic behavior of mimulone might involve a combination of direct receptor interaction and modulation through other pathways or coregulators.

An interesting observation is that the lower of the two concentrations tested (2.5 μM) achieved more activation of the PPARγ signaling pathway than the higher concentration (5 μM). A similar effect was observed for diplacone. A study by Barthel et al. showed the concentration‐dependent influence of troglitazone on the insulin‐induced expression of fatty acid synthase and the activity of protein kinase B in 3T3‐L1 adipocytes. Is found increased activity at low concentrations (250 nM) and inhibitory effects at higher concentrations (≥1 µM). This biphasic response suggests that low concentrations of the compound enhance the activation of the pathway, whereas higher concentrations may inhibit it. The mechanism likely involves the optimal activation of signaling pathways at lower doses and the inhibition of potential feedback or off‐target interactions at higher doses.^[^ ^32^ ^]^ These findings are comparable to our observations with mimulone and diplacone, where the lower concentration (2.5 μM) exhibited superior PPARγ activation compared to the higher concentration (5 μM).

### 2.4 PPARγ Reporter Assay with Mimulone Incorporated into Liposomes

The main limitations of the wider use of flavonoids in therapy include limited solubility, bioavailability, and metabolic stability.^[^ ^33^ ^]^ Encapsulation into nanoparticles represents a promising option to improve these characteristics of flavonoids. Following the identification of mimulone efficacy in activating the PPARγ pathway, our next step was to encapsulate the test substances into various nanoparticle systems. Ones of the most versatile carriers are liposomes. In a previous study by Brezani et al., various types of nanocarriers, including cationic, anionic, and pegylated liposomes meant to improve the solubility and bioavailability of poorly soluble lipophilic compounds of macasiamenene F were tested. The formulation of neutral liposomes composed of DC‐CHOL/CHOL/EPC/DSPE‐PEG 2000 showed high cellular uptake and other desirable properties on various cell lines including THP‐1 and THP‐1‐XBlue‐MD2‐CD14 monocytes and BV‐2 microglia while keeping the anti‐inflammatory properties of macasiamenene F.^[^ ^34^ ^]^

**Figure 5:**
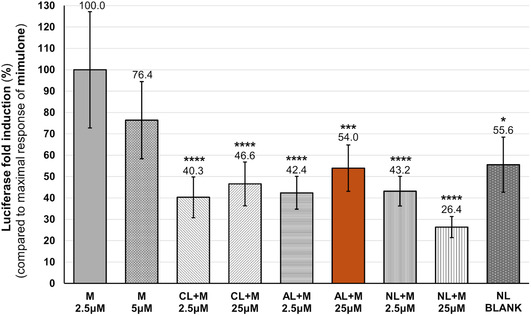
PPARγ pathway activation of mimulone (M) encapsulated within liposomes. Luciferase activity was measured by the CALUX reporter gene assay, calculated as the fold induction compared to the solvent control (DMSO), and expressed as a percentage of the maximal response of mimulone. The liposomes were divided into groups according to their different zeta potentials: cationic (CL), anionic (AL), and neutral (NL). Values refer to the specific concentrations of the encapsulated mimulone; the BLANK samples were prepared without mimulone. The results are expressed as the mean ± SEM for three independent experiments measured in tetraplicate. Statistical analysis is based on the Kruskal Wallis test followed by the Bonferroni correction for multiple tests. Asterisks indicate a significant difference from the maximal response of mimulone: *p < 0.05; **p < 0.01; ***p < 0.001; and ****p < 0.0001.

In this study, we tested anionic, cationic, and neutral liposomes and two concentrations of the encapsulated compound: one was identical to the active concentration of the pure substance (i.e., 2.5 μM), the other was ten times as high (25 μM). AL showed a 2.10‐fold induction of PPARγ activation (AL + 25 μM mimulone, *p* ≤ 0.01 compared to the negative control), indicating effective delivery but with a significant reduction in maximal response compared to free mimulone. The experiments aimed to find the composition of the nanoparticle system that would preserve the maximum biological activity of mimulone, that is, the luciferase activity should not differ significantly from free mimulone. The results with all groups of liposomes are summarized in **Figure** **5**, with luciferase activity expressed as a percentage of the fold change relative to the maximal response of mimulone. The best anionic liposome formulation retained 54% of the biological activity of mimulone. Cationic and neutral liposomes also activated PPARγ, but their efficacy was lower than that of AL and free mimulone.

One plausible explanation for the reduced efficacy of liposome‐encapsulated mimulone is the strong binding of mimulone within the liposome membrane. Due to its smaller and more linear structure (chalcone backbone), mimulone can intercalate easily into the lipid bilayer. The hydroxyl groups present can form hydrogen bonds with the polar head groups of lipids. Similar findings are presented in the work of Wesołowska et al., where prenylated chalcones and flavanones from the common hop were shown to intercalate into DPPC bilayers, decreasing the melting temperature and demonstrating strong interactions with the lipid bilayer. The magnitude of the induced effect correlated with the lipophilicity and structure of the compounds, supporting the idea that smaller and more linear structures can intercalate more easily into lipid bilayers.^[^ ^35^ ^]^ Furthermore, the research by Johnston et al. highlights how the structural characteristics of liposomal constituents, including the presence of cholesterol, can modulate the fluidity and stability of the membrane, impacting the encapsulated bioavailability of the drug.^[^ ^36^ ^]^ Experimental studies performed by Kerdudo et al. studied the encapsulation of naringenin and rutin in liposomes. In the case of naringenin, a nonprenylated flavonoid, a significant portion was absorbed on the surface of the liposomes rather than encapsulated. This study also indicated that small flavonoids interact through hydrogen bonding with the head groups on the upper part of the phosphatidylcholine membrane.^[^ ^37^ ^]^ Sadžak et al. published a study on the interaction of flavonoids with lipid bilayers, showing that the hydroxyl groups of flavonoids can form hydrogen bonds with the polar head groups of lipids.^[^ ^38^ ^]^ This interaction was primarily via hydrogen bonds, which is consistent with the proposed mechanism for mimulone. We investigated this hypothesis using DSPC as the model lipid bilayer to investigate the effect of the mimulone behavior of the phospholipid membrane. The results indicate strong interactions between mimulone and the lipid bilayer. Specifically, the incorporation of mimulone into DSPC liposomes led to significant shifts in the transition temperature of the lipid bilayer. For instance, the *T*_m_ of the pure DSPC bilayer was 54.4 ± 0.3 °C, whereas the presence of mimulone at various concentrations caused a decrease in *T*_m_, with the most pronounced effect observed at the highest concentration of 510 µg mL^−1^, where *T*_m_ decreased to 50.8 ± 0.1 °C. This decrease in *T*_m_ suggests that mimulone strongly interacts with the lipid bilayer, disrupting its structure and reducing thermal stability. (For more information about differential scanning calorimetry (DSC) thermograms, please see Appendix A, Figure S6, Supporting Information). These interactions likely involve the intercalation of mimulone within the lipid bilayer, facilitated by its chalcone backbone and the ability to form hydrogen bonds with the lipid head groups. Such interactions can immobilize mimulone within the membrane, limiting its release and reducing its bioavailability and efficacy. To further investigate this aspect, we employed confocal microscopy to examine the entry of fluorescently labeled liposomes into cells. After 8 h of incubation, we observed a pronounced fluorescence signal from the labeled particles within the cells, indicating successful cellular uptake of the liposomes (Figure 1). Interestingly, there was no significant difference in cellular uptake among the test liposomes with varying surface charges. We used cationic liposomes as a positive control, given that the positively charged surface is expected to interact more readily with the negatively charged cell membrane theoretically facilitating a higher rate of uptake. Kang et al. found that liposomes with different surface charges (cationic, neutral, and anionic) showed time‐dependent uptake via specific endocytic pathways. In glioblastoma cells, cationic and AL were primarily taken up via macropinocytosis, whereas neutral liposomes used caveolae‐mediated endocytosis. In fibroblast cells, all liposomes entered via clathrin‐mediated endocytosis. Despite this, the uptake of cationic liposomes was comparable to that of neutral and AL.^[^ ^39^ ^]^ This uniformity in the rates of uptake further supports the notion that the strong interaction between the liposomal lipids and mimulone hinders its release, irrespective of the surface charge of the liposome, thereby reducing its efficacy.

Villegas et al. successfully incorporated four prenylated flavonoids into polymeric lipophilic nanoparticles, whose negative zeta potential values ensured good stability of the nanoparticles. It has been previously reported that compared to liposomes, polymers have a lower loading capacity but are more stable and allow for more controlled properties of release.^[^ ^40^ ^]^ One of the test substances, 5,7‐dihydroxy‐6‐prenylflavanone, which differs from mimulone only by the absence of the C‐4′ hydroxyl group, demonstrated the best results among the TC in release and skin permeation studies and showed anti‐inflammatory activity. Our findings are consistent with these results, indicating a higher efficacy of nanoparticles with negative zeta potential. Additionally, the study reported a smaller drug release rate (50%) from nanoparticles for the compound discussed. This finding could correlate with our results concerning reducing the maximal response of mimulone incorporated into liposomes to about half the efficacy of the free compound.^[^ ^41^ ^]^

### 2.5 PPARγ Reporter Assay with Mimulone Incorporated into HP‐β‐Cyclodextrins

**Figure 6:**
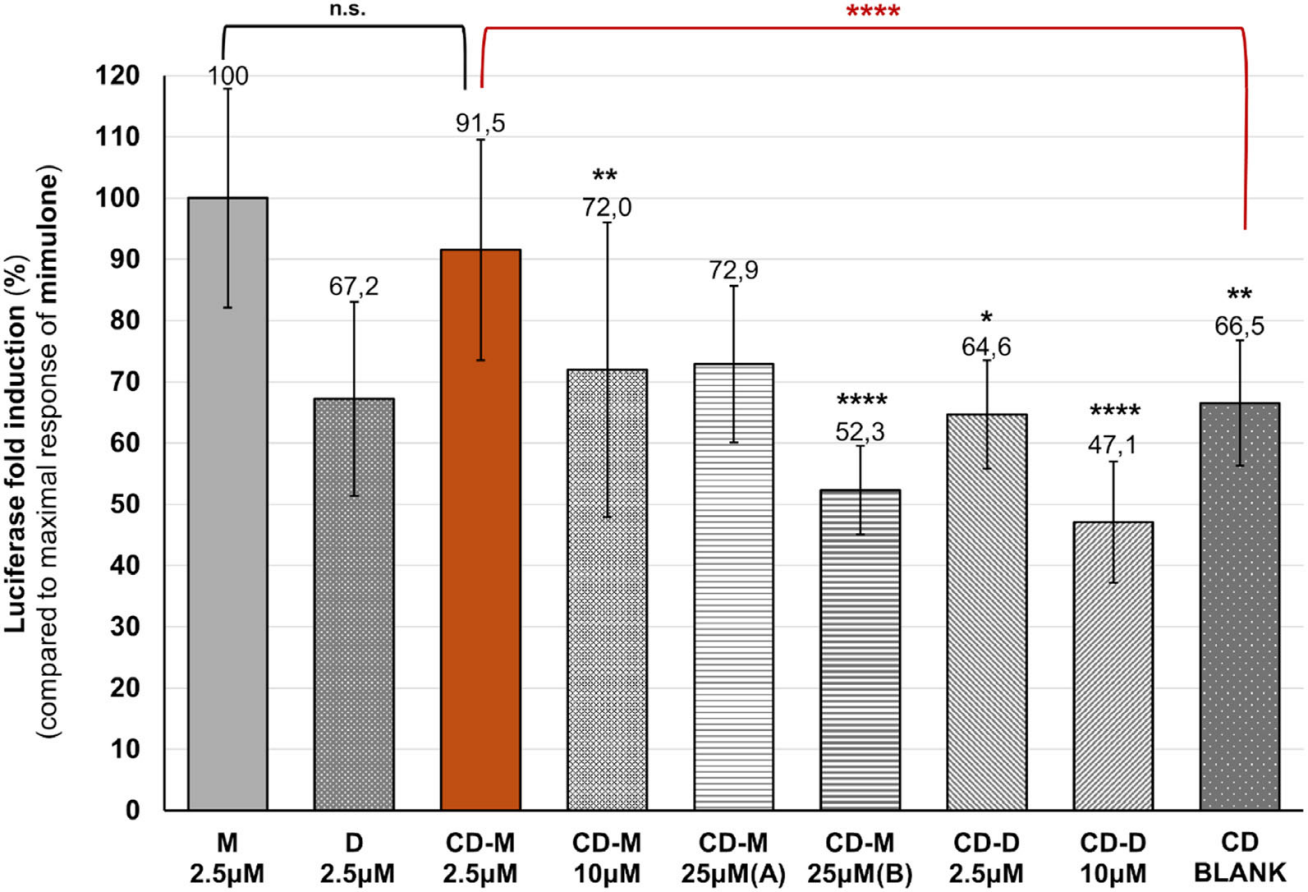
Activation of the PPARγ pathway of mimulone (M) and diplacone (D) encapsulated into HP‐β‐cyclodextrins. The luciferase activity was measured by the CALUX reporter gene assay and expressed as percentages of the fold change relative to the maximal response of mimulone. CD refers to HP‐β‐cyclodextrins, the letters (M, D) indicate specific combinations with the test compound. Values refer to the exact concentrations of mimulone used; the BLANK samples were prepared without mimulone. Two CD‐M 25 μM combinations differ in CD composition: Sample A contained a CD concentration of 10 mg mL^−1^, which was insufficient to encapsulate the active compound at 25 μM. To address this problem, the CD concentration was increased to 100 mg mL^−1^ in sample B. The results are expressed as the mean ± SEM for three independent experiments measured in tetraplicate. Statistical analysis is based on the Mann–Whitney U test and the Kruskal Wallis test, followed by the Bonferroni correction for multiple tests. Asterisks indicate a significant difference from the maximal response of mimulone: *p < 0.05; **p < 0.01; ***p < 0.001; and ****p < 0.0001.

Considering the significant loss of biological efficacy of mimulone in liposomes, the next step was transition to another nanoparticle system with different parameters expected to encapsulate the lipophilic substance in the nanoparticle cavity. HP‐β‐CDs were selected for further testing. Previous findings confirmed that lipophilic compounds are entrapped in the hydrophobic cavity of CDs, which increases their solubility and stability. However, CDs have a limited ability to accommodate compounds due to the fixed size of their cavity.^[^ ^42^ ^]^ In our experiments, HP‐β‐CDs proved to be a more effective nanoparticle system for maintaining the biological efficacy of mimulone than liposomes. The most effective combination (HP‐β‐CD + 2.5 μM mimulone) demonstrated only a 10.5% loss in the maximal mimulone response (the fold induction of the PPARγ activation measured was not statistically different from that of mimulone dissolved in DMSO) and notably showed a significantly different response compared to “empty” cyclodextrins (the CD blank, without an active substance incorporated). This combination also significantly improved the activation of the test pathway compared to the negative control—DMSO (*p* ≤ 0.05). A summary of the experiments to determine the activation of the PPARγ pathway of the TC incorporated into cyclodextrins is shown in **Figure** **6**. The luciferase activity is expressed as percentages of the fold change relative to the maximum response of mimulone.

Cyclodextrins are known for their ability to form inclusion complexes with various compounds, including flavonoids, which enhances their solubility, stability, and bioavailability. Some studies have shown that cyclodextrin complexes can provide controlled release and better protection for encapsulated substances.^[^ ^43,^, ^44^ ^]^ Several studies have indicated that cyclodextrins might be more effective than liposomes for certain applications. For example, in the study by Azzi et al., the flavonoid quercetin encapsulated in cyclodextrins showed greater stability and solubility than liposomal formulations.^[^ ^45^ ^]^ In addition, a study by Yao et al. on the flavonoid glycoside didymin demonstrated that its inclusion complex with β‐cyclodextrin and hydroxypropyl‐β‐cyclodextrin significantly improved its water solubility and bioavailability.^[^ ^46^ ^]^ A study by Costescu et al. provided a comparative analysis of the cavity characteristics of cyclodextrins and small unilamellar liposomes using molecular modeling techniques. The results suggest that cyclodextrins provide a more stable and efficient encapsulation environment for small, hydrophobic molecules, such as flavonoids, compared to liposomes.^[^ ^47^ ^]^ An interesting comparison to our results is provided by a study that evaluated the bioavailability of hesperetin in various nanoparticles, including coated nanoliposomes and cyclodextrins.^[^ ^42^ ^]^ Hesperetin ((S)‐5,7‐dihydroxy‐2‐(3‐hydroxy‐4‐methoxyphenyl)chroman‐4‐one) is also a flavanone, differing from mimulone in the substitution pattern on the B‐ring (3′‐hydroxy‐4′‐methoxy) and the absence of a prenyl group at the C‐6 position. The study noted that CDs retained 66% of hesperetin after intestinal digestion, suggesting moderate protective efficiency compared to maltodextrin (MD), but less than the coated nanoliposomes, which retained 76% of hesperetin postdigestion and exhibited the highest hesperetin transfer rate through the intestinal epithelium. Cyclodextrins, although effective in enhancing solubility, were reported to be less protective under gastrointestinal conditions compared to robust double‐coated liposomes.^[^ ^42^ ^]^ The difference with our findings may be attributed to the lower lipophilicity of hesperetin compared to mimulone, which likely results in hesperetin being less prone to hydrophobic interactions and binding within the phospholipid bilayer of the nanoparticle, and thus, more easily released from the structure.^[^ ^42^ ^]^

## 3 Conclusion

This study evaluates the PPARγ agonistic potential of mimulone and diplacone, two geranylated flavanones from *P. tomentosa*, and their encapsulation in various nanoparticle systems to enhance bioavailability and stability. Our results demonstrate that mimulone dissolved in DMSO activates the PPARγ signaling pathway, showing a 2.97‐fold and 3.9‐fold increase in luciferase activity at concentrations of 5 and 2.5 μM, respectively. Diplacone, however, exhibited significant cytotoxicity, with an average cell viability of about 10% at 10 μM.

Encapsulation into anionic, cationic, and neutral liposomes resulted in a significant reduction in the biological activity of the active compound. The most effective liposomal formulation, AL, preserved only 54% of the activity of mimulone.

In contrast, HP‐β‐CDs emerged as a superior nanoparticle system for maintaining the biological activity of both flavonoids. The most effective combination (HP‐β‐CD + 2.5 μM mimulone) showed only a 10.5% loss of maximal mimulone response, with PPARγ activation comparable to that of DMSO‐dissolved mimulone. Cyclodextrin encapsulation also significantly improved the viability profile of diplacone, maintaining cell viability at around 100%, in stark contrast to the free form of the compound. The enhanced performance of HP‐β‐CDs could be attributed to their ability to form stable inclusion complexes with hydrophobic molecules, thereby improving solubility, stability, and controlled release. These findings suggest that cyclodextrin‐based delivery systems effectively address the solubility and stability challenges associated with flavonoid therapy and offer a promising approach to improving the bioavailability and therapeutic efficacy of these compounds.

## 4 Experimental Section

4.1

4.1.1

##### Chemicals

Rosiglitazone (CAS no: 122,320‐73−4), Dimethyl sulfoxide (CAS no:67‐68‐5, DMSO, 99.9%), Phosphate buffered saline (PBS), Dulbecco's Modified Eagle Medium with Ham's Nutrient Mixture F‐12 (1:1) (DMEM/F12) without phenol red, Nonessential amino acids (NEAA), Fetal Bovine Serum (FBS), and Charcoal Stripped Fetal Bovine Serum (CS‐FBS) were purchased from Merck (KGaA, Darmstadt, Germany); Penicillin‐Streptomycin Solution 100X and trypsin was obtained from Biosera (Cholet, France); DMEM/F‐12 with GlutaMAX supplement was supplied by Thermo Fisher Scientific (Waltham, USA); and chloroform (p.a. 99.8%) by Sigma Aldrich, CZ. Lipids L‐α‐phosphatidylcholine (EPC), 3β‐[N‐(N’,N’‐dimethylaminoethane)‐carbamoyl]cholesterol (DC‐cholesterol), POPG (1‐palmitoyl‐2‐oleoyl‐sn‐glycero‐3‐phospho‐(1‐rac‐glycerol)), DSPC (1,2‐distearoyl‐sn‐glycero‐3‐phosphocholine), and cholesterol were purchased from Avanti Polar Lipids (Alabaster, U.S.). 1,2‐distearoyl‐sn‐glycero‐3‐phosphoethanolamine‐N‐[amino (polyethylene glycol)‐2000] (DSPE‐PEG 2000) was obtained from Nof Corporation (Japan). 2‐Hydroxypropyl‐β‐cyclodextrin (HP‐β‐CD) was purchased from Sigma–Aldrich (CZ; product number H107, CAS: 128446‐35‐5). The average molecular weight was 1396 Da, with a degree of substitution around 0.6 and a purity ≥98%. The compound was provided as a white powder.

##### TC

The TC were isolated from unripe fruits of *P. tomentosa* Steud. (Paulowniaceae) and characterized at the Faculty of Pharmacy, Masaryk University Brno, Brno, Czech Republic.^[^ ^48^ ^,^ ^49^ ^]^ high‐performance liquid chromatography analysis verified that the purity of each test compound exceeded 95%. All of the TC were dissolved in DMSO, with the final DMSO concentration in the cellular assays set at 0.1% (v/v).

##### Preparation of Nanoparticles

Nanoliposomes were prepared using the film hydration method with the following lipids: L‐α‐phosphatidylcholine (EPC), DC‐cholesterol, POPG, DSPE‐PEG 2000, and cholesterol. The liposomal formulations used in this study were based on a previously optimized composition reported by Brezani et al.^[^ ^34^ ^]^ The required amounts of lipids based on **Table** **2**, with or without the active compound (mimulone, at final concentrations of 51 and 204 µg mL^−1^), were dissolved in chloroform in a round‐bottom flask (the total lipid concentration was 5 mg mL^−1^). The solvent was removed using a rotary vacuum evaporator at 55 °C, which is above the highest transition temperature of the lipid mixture. After the removal of the solvent, the resulting thin film was rehydrated with an aqueous solvent (PBS buffer, pH 7.4). Liposomes were homogenized by membrane extrusion through a 200 nm polycarbonate filter (Whatman Nuclepore, Sigma Aldrich, CZ) using a manual extruder (LiposoFast, Avestin, Canada).

**Table 2:** Summary of individual liposomal compositions and corresponding mol%.

Liposomes	Compositions (mol%)
Neutral/PEG	EPC/Chol./DC‐Chol./DSPE‐PEG‐2000 (55/25/15/5)
Anionic	EPC/POPG (70/30)
Cationic	EPC/DC‐Chol (80/20)

Cyclodextrin nanoparticles loaded with mimulone were prepared using the following method. An appropriate amount of HP‐β‐CD was dissolved in phosphate‐buffered saline (PBS, pH 7.4) at a concentration of 10 mg mL^−1^ and/or 100 mg mL^−1^. Separately, mimulone was dissolved in ethanol (EtOH). Under constant stirring, the mimulone solution was added dropwise into the heated (45 °C) HP‐β‐CD solution. The final concentrations of mimulone were 51 and 204 µg mL^−1^, with a volume ratio of 1:1. Based on the molecular weights of HP‐β‐CD and mimulone the resulting guest/host molar ratios were ≈0.007, 0.028, and 0.007, respectively with strong molar excess of the carrier. The mixture was stirred for an additional 2 h to ensure the complete formation of nanoparticles and the encapsulation of mimulone. After this reaction, EtOH was evaporated under reduced pressure (400 PSI, 45 °C), using a rotary vacuum evaporator, and the solution was filtered through a 0.22 µm filter.

##### Characterization of Nanoparticles

The size, polydispersity, and concentration of the nanoparticles were determined using multiangle DLS (MADLS) (Zetasizer Nano ZSP, Malvern, Great Britain). Approximately 50 µL of the sample was placed in a low‐volume quartz batch cuvette ZEN2112 (Malvern Panalytical Ltd, Malvern, UK) and measured using the MADLS technique with a Zetasizer Ultra (Malvern Panalytical Ltd, UK) at a constant temperature of 25 °C. The device was equipped with a HeNe Laser (633 nm) and three detectors set at the angles: 173° (backscatter), 90° (side scatter), and 13° (forward scatter). The zeta (*ζ*) potential of the nanoliposomes was determined using the electrophoretic light scattering method (Zetasizer Nano ZSP, UK). Approximately 800 µL of the sample was placed in a folded capillary zeta cell DTS1070 (Malvern Panalytical Ltd, Malvern, UK). Analysis was performed using the monomodal mode at a constant temperature of 25 °C. The data were evaluated using ZS Xplorer software, version 3.50 (Malvern Panalytical Ltd, UK). The measured values are reported as the mean value (*n* = 3) ± the standard deviation (Table 1).

##### In Vitro Cellular Uptake

To evaluate the uptake of the liposomes by PPAR‐γ2 CALUX cells, liposomes were labeled with a fluorescent probe. The fluorescently labeled liposomes were prepared as described previously, with the addition of the fluorescent dye 1,2‐dioleoyl‐sn‐glycero‐3‐phosphoethanolamine‐N‐(lissamine rhodamine B sulfonyl) (Lis‐Rhodamin‐PE) at a final concentration 0.1 mol%. The wavelengths for the excitation and emission maximum for this probe were 560 and 583 nm, respectively. The fluorescently labeled liposomes (50 µg mL^−1^) were incubated with the target cells under standard culture conditions. The cells were seeded to achieve ≈50% confluence before proceeding with further experimental steps. After incubation (8 h) in µ‐Slide 8 Well (IBIDI), the cells were washed using PBS buffer to remove any unbound liposomes and fixed for confocal microscopic analysis. The uptake of the fluorescent liposomes by the cells was visualized using a Leica TCS SP8 MP confocal fluorescence microscope (Leica Microsystems. DE).

##### EE

The EE was calculated from the UV–VIS data obtained using a DAD spectrometer (Specord S600, Analytik Jena, DE). For the construction of the calibration curve, the required amount of mimulone was dissolved in absolute ethanol (EtOH) and diluted to concentrations ranging from 1 to 0.003 mg mL^−1^. Approximately 400 µL of each sample was placed into a UV Quartz SUPRASIL Semi‐Micro Cell with a 10 mm path length and measured in the range from 200 to 380 nm at a constant temperature of 25 °C. The concentration of mimulone in the nanoparticles was determined using the established calibration curve. All samples were measured against blank samples, which consisted of either liposomes or cyclodextrins without the active compound. The spectrum of the blank was subtracted from the sample spectra to ensure accurate analysis. The EE was calculated using the following formula

(1) $$\mathrm{EE}\left(\%\right)=\frac{\text{Concentration of encapsulated mimulone}}{\text{Theoretical concentration of mimulone}}\times 100$$

##### DSC Analysis

The lipid transition temperature (*T*_m_) and the influence of the active compound, mimulone, on the model of lipid membrane were analyzed using a MicroCal PEAQ‐DSC Automated system (Malvern Panalytical Ltd, Malvern, UK). A sample volume of 350 µL was used for the analysis, and PBS buffer at pH 7.4 served as the reference. DSPC liposomes were prepared using the hydration of lipid film method with a total lipid concentration of 5 mg mL^−1^ and various concentrations of mimulone: 0 µg mL^−1^ (blank), 51, 204, and 510 µg mL^−1^. Each sample was measured in duplicate. The samples were loaded into the DSC cell, and the reference cell was filled with PBS buffer. The DSC scans were conducted from 5 to 120 °C at a rate of 120 °C per hour. Data collection and analysis were performed using MicroCal PEAQ‐DSC software, version 2.21, to determine the transition temperatures.

##### Cell Culture

The PPAR‐γ2 CALUX cells, obtained from BioDetection Systems BV (Amsterdam, the Netherlands), were derived from human osteosarcoma U2OS cells and had been stably transfected with a PPAR‐γ2 expression vector along with a firefly luciferase reporter construct controlled by the peroxisome proliferator responsive element.^[^ ^50^ ^]^ These cells were cultured in DMEM/F12 GlutaMAX medium enriched with 7.5% fetal bovine serum, 1% nonessential amino acids, 100 U mL^−1^ penicillin, and 100 mg mL^−1^ streptomycin. To ensure the selection pressure, G418 (Roche, Mannheim, Germany) at a final concentration of 200 µg mL^−1^ was added once a week. The cells were incubated at 37 °C in a humidified environment with 5% CO_2_.

##### Cell Viability Testing (Cytotoxicity Assay)

The viability of PPAR‐γ2 CALUX cells was measured using the cell proliferation reagent WST‐1 (Roche, Basel, Switzerland) according to the manufacturer's manual, as reported previously.^[^ ^51^ ^]^

##### Reporter Gene Assays

The ability of flavanones to stimulate PPAR‐γ2‐driven luciferase expression was evaluated by measuring the luciferase activity in PPAR‐γ2 CALUX reporter cells. PPAR‐γ2 CALUX cells were seeded into 96‐well tissue culture‐treated microtiter plates (TPP, Switzerland) at a density of 10,000 cells per well in 100 μL of assay medium (DMEM/F12 without phenol red, supplemented with 5% charcoal‐stripped FBS and 1% NEAA, all from Merck KGaA, Darmstadt, Germany). After a 24‐h incubation period to allow cell adherence and the formation of a confluent monolayer, the central 60 wells were treated with the TC at specified concentrations in an assay medium for an additional 24 h. Each concentration was tested in a tetraplicate. The final DMSO concentration in the medium was maintained at 0.1%. Following this exposure, the cells were examined microscopically for cytotoxic effects. The medium was then removed, the plates were frozen at −80 °C for 15 min, and the cells were lysed with 30 μL of a lysis buffer containing 25 mM Tris buffer (pH 7.8), 2 mM dithiothreitol, 2 mM 1,2‐diaminocyclohexane‐tetraacetic acid, 10% glycerol, and 1% Triton X‐100, procured from BDS (Amsterdam, Netherlands). The luciferase activity was quantified using a luminometer (FLUOstar Omega microplate reader, BMG Labtech, Germany) after adding 100 μL of flash mix (20 mM tricine, 1.07 mM (MgCO_3_)_4_ Mg (OH)_2_, 2.67 mM MgSO_4_, 0.1 mM EDTA, 2.0 mM dithiothreitol, 470 μM luciferin, 5.0 mM ATP) per well. The flash mix was also obtained from BDS (Amsterdam, Netherlands). Luciferase activity was reported as relative light units per well.

##### Statistical Analysis

The experimental data were processed in Excel (Microsoft). Statistical analyses were carried out using IBM SPSS Statistics for Windows, software version 26.0 (Armonk, NY, USA). The data were graphed as the mean ± SEM. Comparisons between groups were made using the Mann–Whitney U test and/or a Kruskal Wallis test followed by the Bonferroni correction for multiple tests.

## AI Statement

During the preparation of this work the author(s) used ChatGPT 4 in order to improve the readability and language of the research paper. After using this tool, the authors reviewed and edited the content as needed and take full responsibility for the content of the publication.

## Conflict of Interest

The authors declare no conflict of interest.

## Supporting information

Supplementary Material

## Data Availability

The data that support the findings of this study are available from the corresponding author upon reasonable request.
