# Supplementary material for: PPARγ Agonistic Activity of Mimulone and Diplacone Encapsulated in Liposomes and Cyclodextrin Complexes
Source: ChemistryOpen. 2025 Jul 31;14(12):e202500209. doi: 10.1002/open.202500209 (PMC12680580; doi:10.1002/open.202500209)
Supplement: Supplementary file 1 — Supplementary Material [file OPEN-14-e202500209-s001.pdf]

Supporting Information

Table of contents:

1) Figure S.1.
2) Figure S.2.
3) Figure S.3.
4) Figure S.4.
5) Figure S.5.
6) Figure S.6.

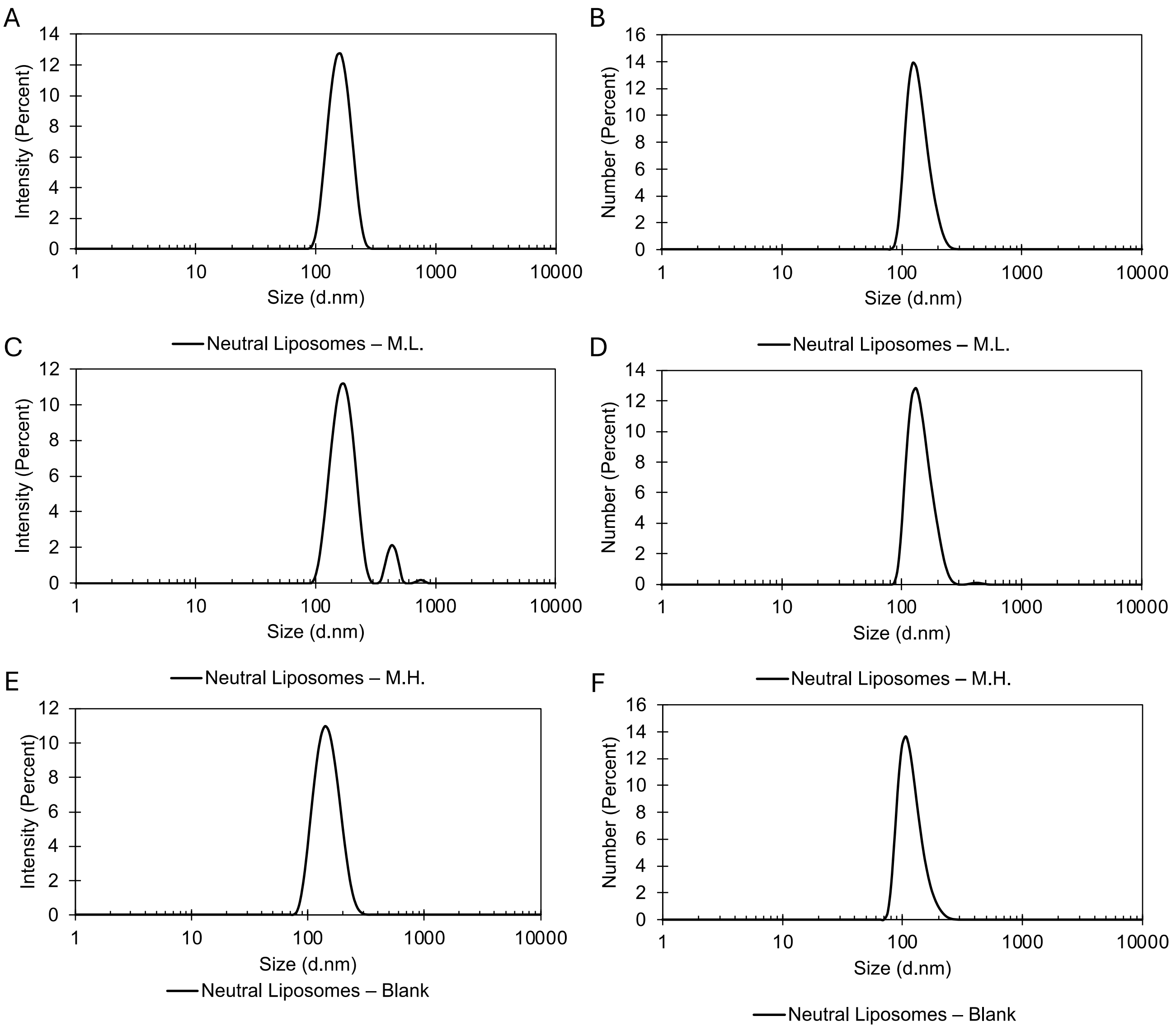

Figure S.1. Intensity and number distribution of Neutral/PEG liposomes composed of EPC/Chol./DC-Chol./DSPE-PEG-2000 (55/40/25/5 mol%). Panels A and B represent neutral liposomes with a low mimulone concentration (M.L.) of 51 µg/mL. Panels C and D represent neutral liposomes with a high mimulone concentration (M.H.) of 204 µg/mL. Panels E and F represent empty, neutral liposomes without mimulone (blank).

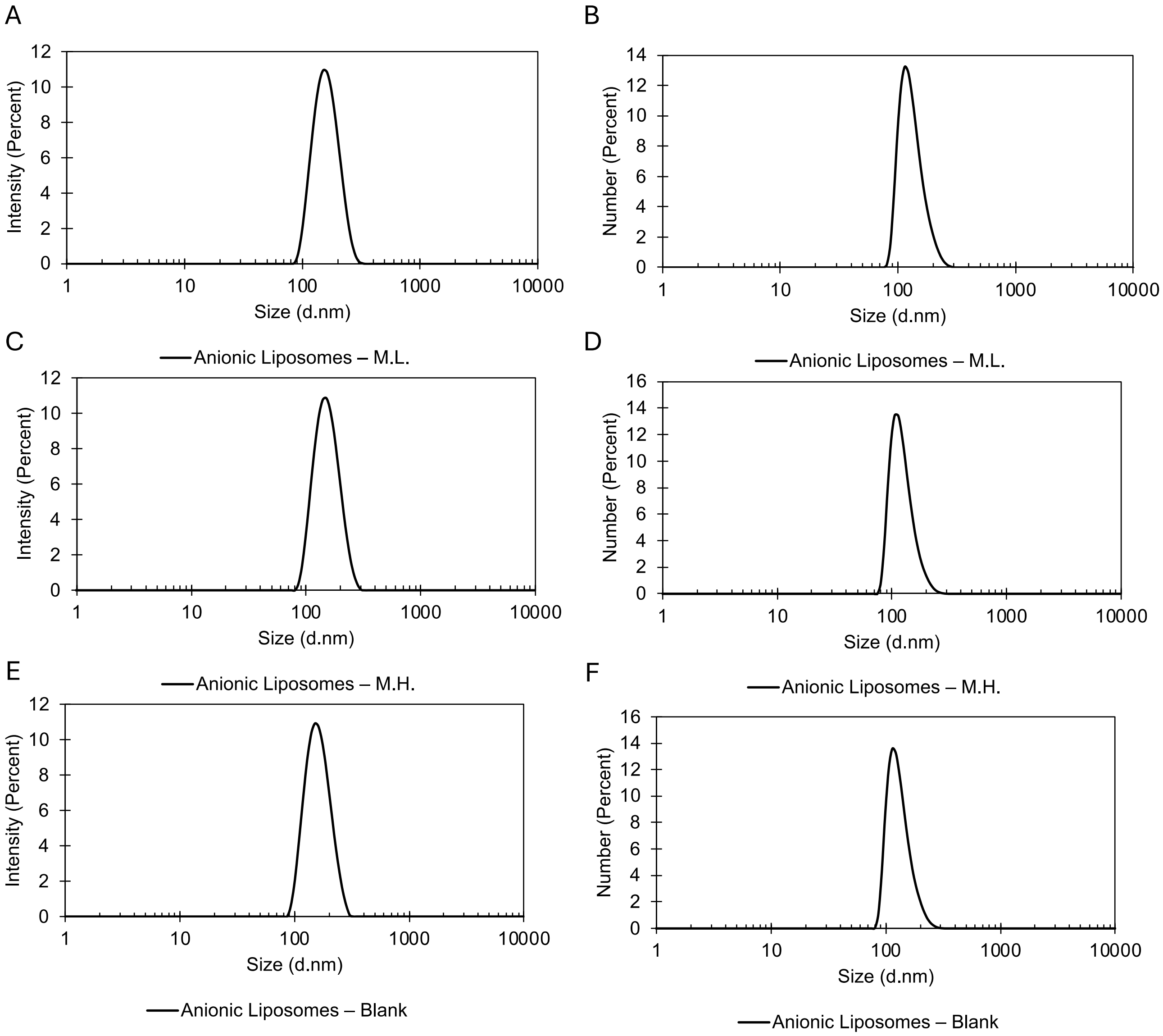

Figure S.2. Intensity and number distribution of anionic liposomes composed of EPC/POPG (70/30 mol%). Panels A and B represent anionic liposomes with a low mimulone concentration (M.L.) of 51 µg/mL. Panels C and D represent anionic liposomes with a high mimulone concentration (M.H.) of 204 µg/mL. Panels E and F represent empty, anionic liposomes without mimulone (blank).

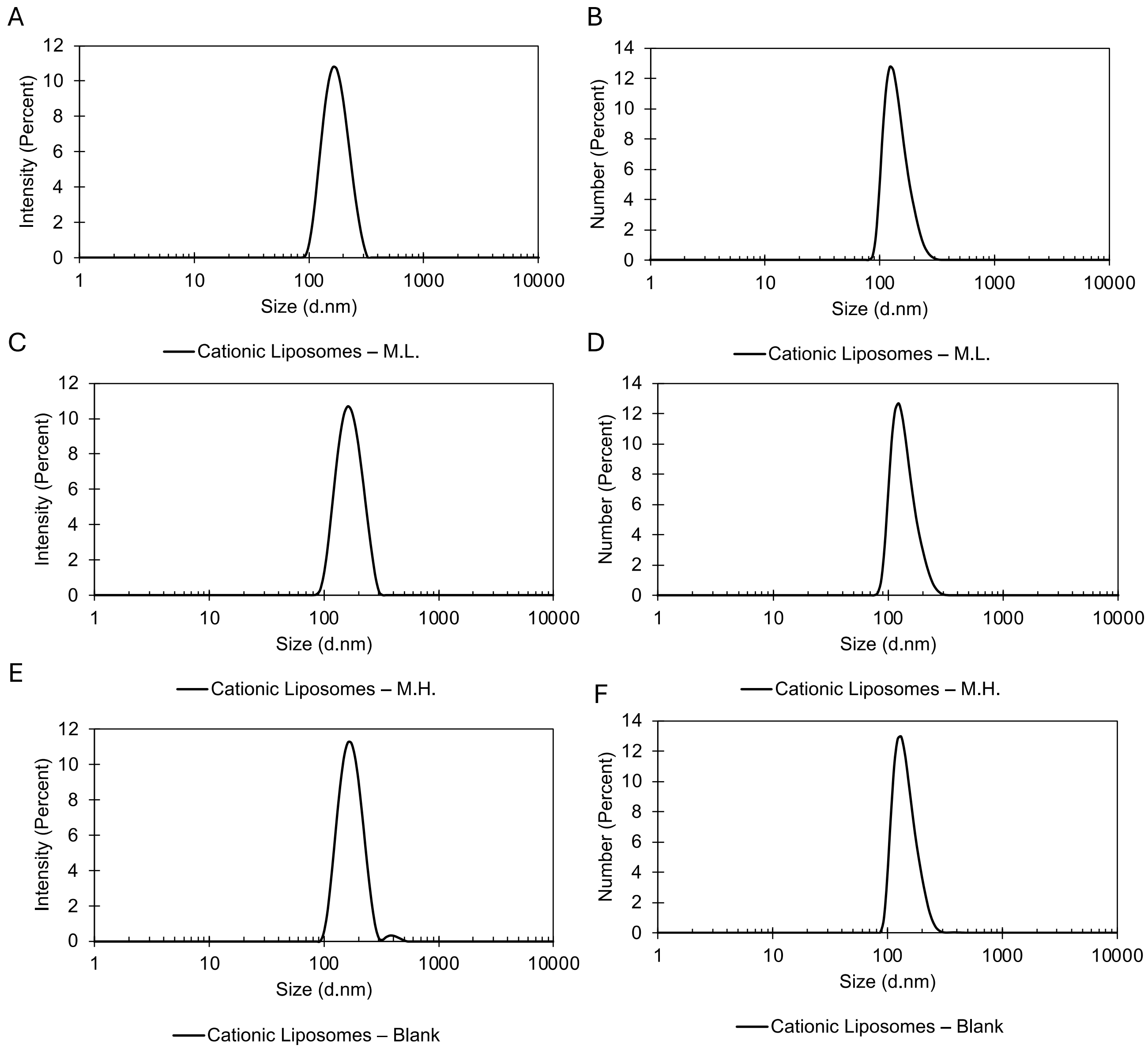

Figure S.3. Intensity and number distribution of cationic liposomes composed of EPC/DC-Chol (80/20 mol%). Panels A and B represent cationic liposomes with a low mimulone concentration (M.L.) of 51 µg/mL. Panels C and D represent cationic liposomes with a high mimulone concentration (M.H.) of 204 µg/mL. Panels E and F represent empty, cationic liposomes without mimulone (blank).

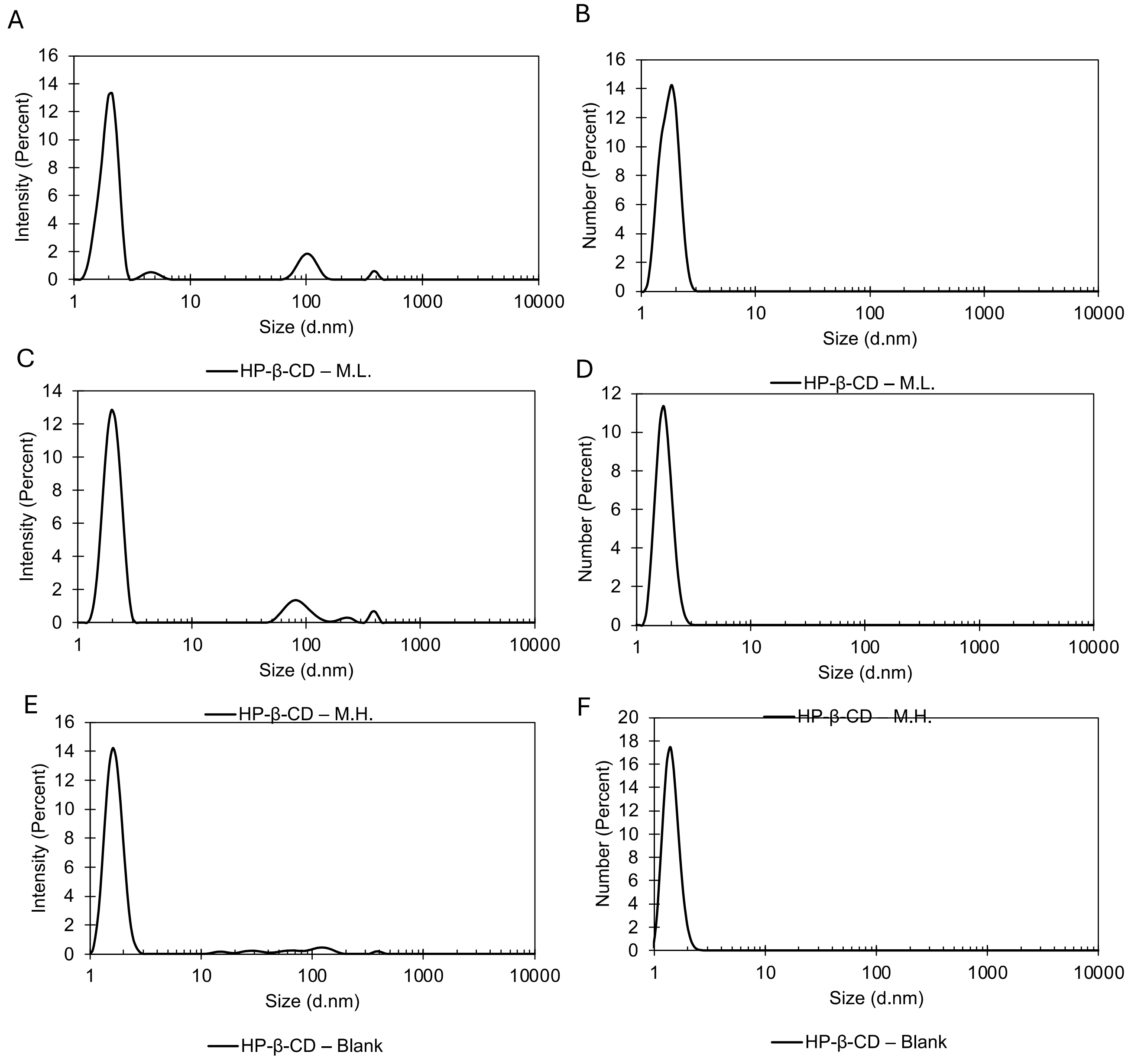

Figure S.4. Intensity and number distribution of HP-β-CD (10 mg/mL). Panels A and B represent HP-β-CD with a low mimulone concentration (M.L.) of 51 µg/mL. Panels C and D represent HP-β-CD with a high mimulone concentration (M.H.) of 510 µg/mL. Panels E and F represent empty HP-β-CD without mimulone (blank).

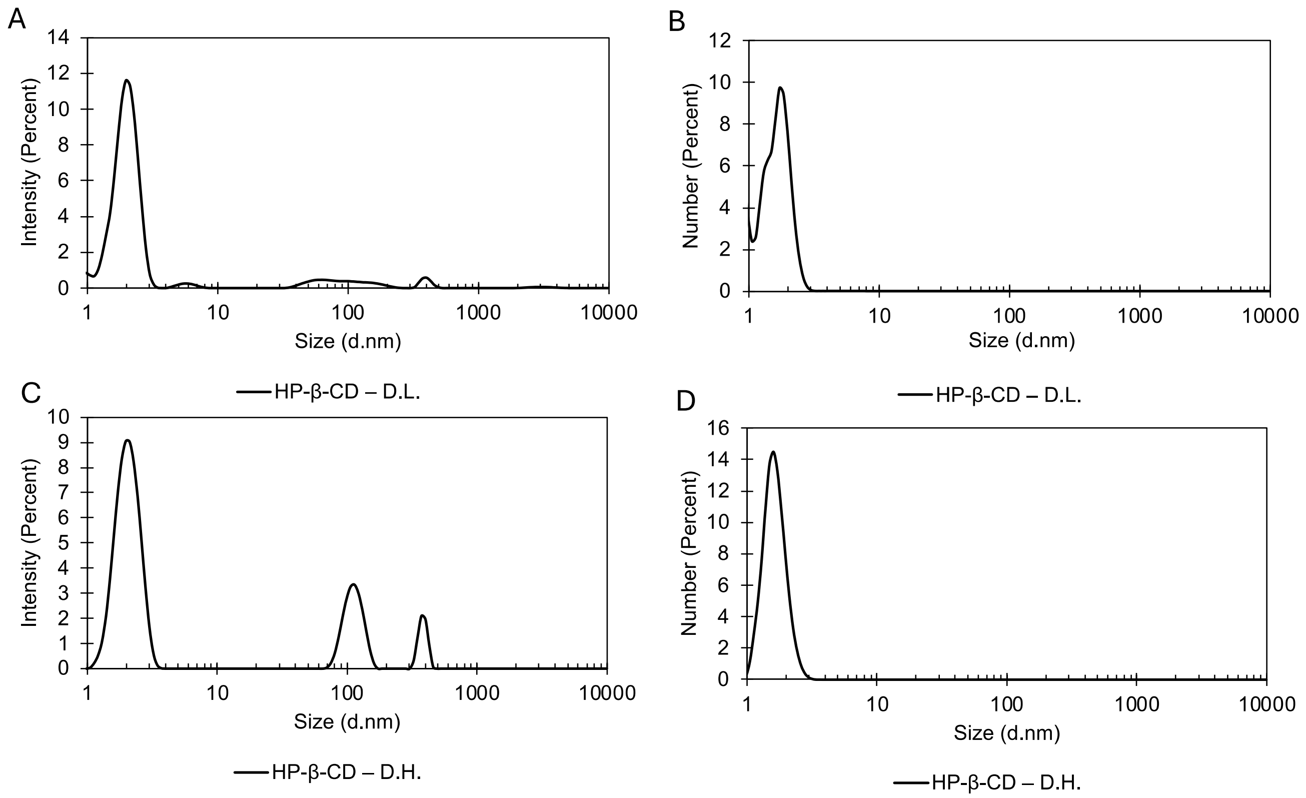

Figure S.5. Intensity and number distribution of HP-β-CD (10 mg/mL). Panels A and B represent HP-β-CD with a low diplacone concentration (D.L.) of 51 μg/mL. Panels C and D represent HP-β-CD with a high diplacone concentration (D.H.) of 510 μg/mL.

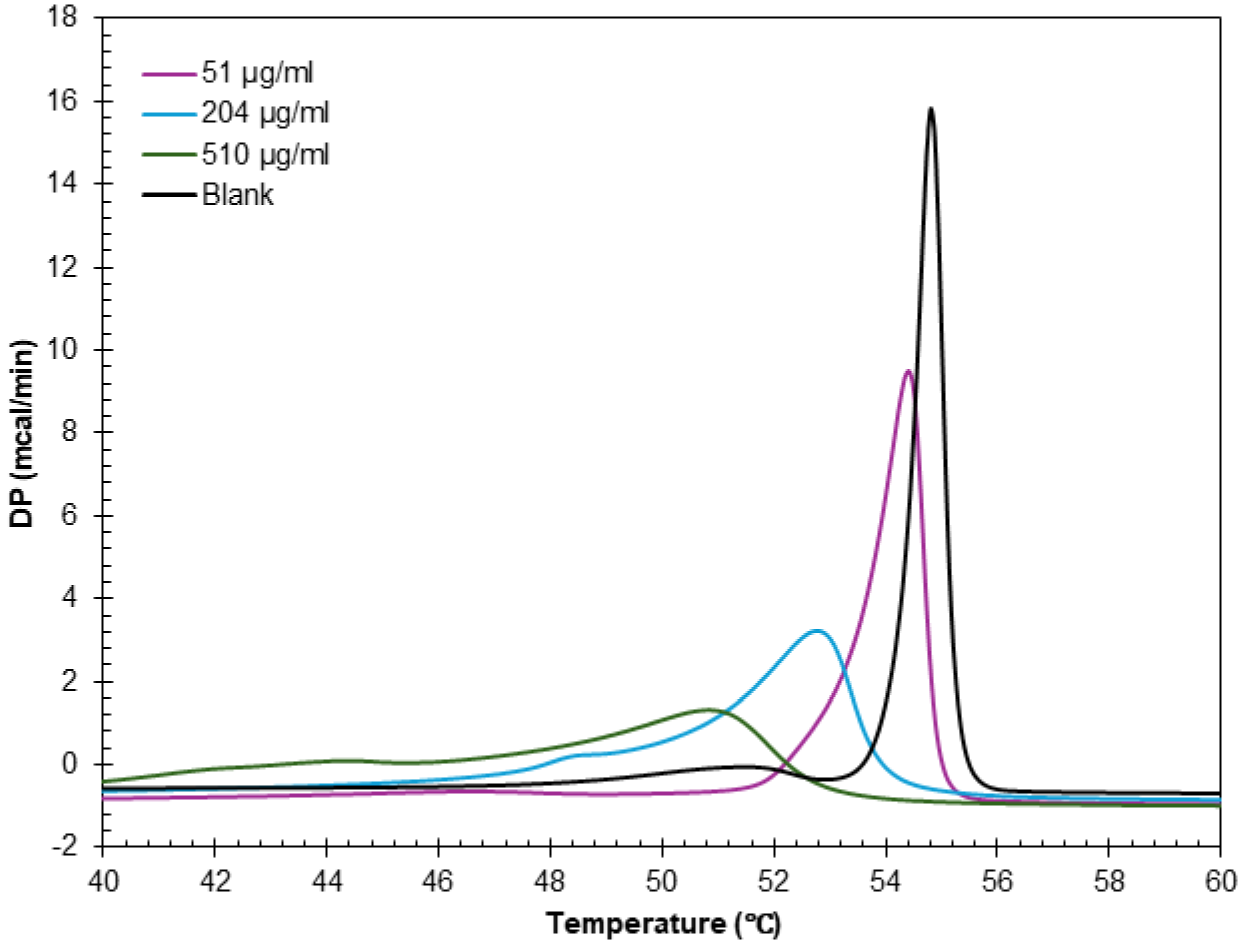

Figure S.6. DSC characterization of the interaction between mimulone and the model lipid membrane. The thermograms show the following data: (black) blank pure DSPC sample with a Tm of 54.4 ± 0.3°C, (blue) DSPC with 51 µg/mL of mimulone and a Tm of 53.9 ± 0.4°C, (yellow) DSPC with 204 µg/mL of mimulone and a Tm of 52.6 ± 0.2°C, and (green) DSPC with 510 µg/mL of mimulone and a Tm of 50.8 ± 0.1°C.
